# Tolerability of maintenance olaparib in newly diagnosed patients with advanced ovarian cancer and a BRCA mutation in the randomized phase III SOLO1 trial

**DOI:** 10.1016/j.ygyno.2021.07.016

**Published:** 2021-08-02

**Authors:** Nicoletta Colombo, Kathleen Moore, Giovanni Scambia, Ana Oaknin, Michael Friedlander, Alla Lisyanskaya, Anne Floquet, Alexandra Leary, Gabe S. Sonke, Charlie Gourley, Susana Banerjee, Amit Oza, Antonio González-Martín, Carol Aghajanian, William H. Bradley, Jae-Weon Kim, Cara Mathews, Joyce Liu, Elizabeth S. Lowe, Ralph Bloomfield, Paul DiSilvestro

**Affiliations:** a University of Milan-Bicocca and IEO European Institute of Oncology IRCCS, Milan, Italy; b Stephenson Cancer Center at the University of Oklahoma, Oklahoma City, OK, United States; c Fondazione Policlinico Universitario A. Gemelli IRCCS Università Cattolica, Rome, Italy; d Vall d'Hebron University Hospital, Vall d'Hebron Institute of Oncology (VHIO), Barcelona, Spain; e University of New South Wales Clinical School, Prince of Wales Hospital, Randwick, Australia; f St Petersburg City Oncology Dispensary, St Petersburg, Russia; g Institut Bergonié, Comprehensive Cancer Centre, Bordeaux, France; h Groupe d'Investigateurs Nationaux pour l'Etude des Cancers Ovariens (GINECO), Paris, France; i Institut Gustave-Roussy, Villejuif, France; j The Netherlands Cancer Institute, Amsterdam, the Netherlands; k Cancer Research UK Edinburgh Centre, Institute of Genetics and Molecular Medicine, University of Edinburgh, Edinburgh, United Kingdom; l The Royal Marsden NHS Foundation Trust, and Institute of Cancer Research, London, United Kingdom; m Princess Margaret Cancer Centre, Toronto, Canada; n Clínica Universidad de Navarra, Madrid, Spain; o Memorial Sloan Kettering Cancer Center, New York, NY, United States; p Froedtert and the Medical College ofWisconsin, Milwaukee, WI, United States; q Seoul National University, Seoul, South Korea; r Women & Infants Hospital, Providence, RI, United States; s Dana-Farber Cancer Institute, Boston, MA, United States; t AstraZeneca, Gaithersburg, MD, United States; u AstraZeneca, Cambridge, United Kingdom

**Keywords:** Olaparib, Ovarian cancer, Tolerability, Safety, Newly diagnosed

## Abstract

**Objectives.:**

In the phase III SOLO1 trial (NCT01844986), maintenance olaparib provided a substantial progression-free survival benefit in patients with newly diagnosed, advanced ovarian cancer and a BRCA mutation who were in response after platinum-based chemotherapy. We analyzed the timing, duration and grade of the most common hematologic and non-hematologic adverse events in SOLO1.

**Methods.:**

Eligible patients were randomized to olaparib tablets 300 mg twice daily (N = 260)or placebo (N = 131), with a 2-year treatment cap in most patients. Safety outcomes were analyzed in detail in randomized patients who received at least one dose of study drug (olaparib, n = 260; placebo, n = 130).

**Results.:**

Median time to first onset of the most common hematologic (anemia, neutropenia, thrombocytopenia) and non-hematologic (nausea, fatigue/asthenia, vomiting) adverse events was <3 months in olaparibtreated patients. The first event of anemia, neutropenia, thrombocytopenia, nausea and vomiting lasted a median of <2 months and the first event of fatigue/asthenia lasted a median of 3.48 months in the olaparib group. These adverse events were manageable with supportive treatment and/or olaparib dose modification in most patients, with few patients requiring discontinuation of olaparib. Of 162 patients still receiving olaparib at month 24, 64.2% were receiving the recommended starting dose of olaparib 300 mg twice daily.

**Conclusions.:**

Maintenance olaparib had a predictable and manageable adverse event profile in the newly diagnosed setting with no new safety signals identified. Adverse events usually occurred early, were largely manageable and led to discontinuation in a minority of patients.

## 1. Introduction

In women with newly diagnosed, advanced ovarian cancer who are in response to first-line platinum-based chemotherapy, maintenance therapy with the poly(ADP-ribose) polymerase (PARP) inhibitor olaparib is approved in the USA, the EU, China, Japan and other countries worldwide for women with a *BRCA1* and/or *BRCA2* mutation (BRCAm) [1-4] and maintenance olaparib plus bevacizumab is approved in the USA, the EU and Japan for women who test positive for homologous recombination deficiency (BRCAm and/or genomic instability) [1,2,5].

Given that following cytoreductive surgery and platinum-based chemotherapy, patients with newly diagnosed, advanced ovarian cancer will receive maintenance olaparib for a planned 2 years in the setting of no or minimal disease, it is important to establish that olaparib does not add a significant safety or toxicity burden. Adverse events (AEs) should be manageable over time and not lead to treatment discontinuation.

In the phase III SOLO1 trial (NCT01844986; GOG-3004), maintenance olaparib provided a substantial progression-free survival (PFS) benefit in women with newly diagnosed, advanced ovarian cancer and a BRCAm who were in response after platinum-based chemotherapy [6]. In the primary analysis, the risk of disease progression or death was significantly reduced by 70% with olaparib versus placebo (hazard ratio 0.30; 95% CI 0.23–0.41; primary endpoint) [6]. With longer-term follow-up, 48.3% of olaparib patients versus 20.5% of placebo patients were progression free at 5 years (Kaplan-Meier estimates) [7]. The safety profile of maintenance olaparib in the newly diagnosed setting [6] was consistent with that previously reported in the relapsed disease setting [8,9].

The current analysis provides further information about the safety and tolerability of maintenance olaparib in women with newly diagnosed, advanced ovarian cancer and a BRCAm in the SOLO1 trial, with a focus on the most commonly reported hematologic and non-hematologic AEs.

## 2. Methods

### 2.1. Study design and patients

The design of the randomized, double-blind, multicenter, phase III SOLO1 study has been reported previously [6]. In brief, eligible patients had newly diagnosed, histologically confirmed, International Federation of Gynecology and Obstetrics (FIGO) stage III–IV, high-grade serous or endometrioid ovarian cancer, primary peritoneal cancer and/or fallopian tube cancer and a BRCAm. Patients with stage III disease had an upfront or interval attempt at optimal cytoreductive surgery and patients with stage IV disease had a biopsy and/or upfront or interval cytoreductive surgery. Patients had received first-line platinum-based chemotherapy and were in clinical complete response (CR) or partial response (PR) [6]. Any persistent toxicities associated with prior chemotherapy (excluding alopecia) were required to have improved to grade ≤1. Patients were required to have a baseline hemoglobin level of ≥10.0 g/dL (with no blood transfusion in the past 28 days), an absolute neutrophil count of ≥1.5 × 10^9^/L, and a platelet count of ≥100 × 10^9^/L. Full eligibility criteria are provided in the Supplementary Appendix.

The trial was performed in accordance with the Declaration of Helsinki, Good Clinical Practice Guidelines and the AstraZeneca policy of bioethics, under the auspices of an Independent Data Monitoring Committee. AstraZeneca was responsible for overseeing the collection, analysis and interpretation of the data. All patients provided written informed consent.

### 2.2. Random assignment and procedures

Within 8 weeks of completing platinum-based chemotherapy, patients were randomized in a 2:1 ratio to olaparib tablets 300 mg twice daily or matching placebo using an interactive voice and web response system. Randomization used a block design with stratification according to the response to platinum-based chemotherapy (clinical CR or PR).

Study treatment continued until investigator-assessed objective radiologic disease progression (modified Response Evaluation Criteria in Solid Tumors [RECIST] version 1.1 criteria), stopped at 2 years in patients who achieved CR or with no evidence of disease, or could continue beyond 2 years in patients with ongoing PR.

AEs were monitored during, and for 30 days after discontinuation of, study treatment and were graded using National Cancer Institute Common Terminology Criteria for Adverse Events (CTCAE), version 4.0. All ongoing AEs at the time of study treatment discontinuation and any new AEs identified during the 30-day safety follow-up period were followed to resolution unless they were considered unlikely to resolve or the patient was lost to follow-up. Follow-up for myelodysplastic syndromes (MDS)/acute myeloid leukemia (AML) and new primary malignancies was actively continued alongside survival follow-up.

Supportive treatment for AEs was administered according to local practice guidelines, with toxicity also managed by dose modification or discontinuation (Supplementary Appendix).

### 2.3. Outcomes

The primary efficacy endpoint in SOLO1 (investigator-assessed PFS according to modified RECIST version 1.1 criteria) has been reported previously [6].

The safety and tolerability of maintenance olaparib was also assessed. The incidence and prevalence of the most common hematologic and non-hematologic AEs were analyzed. Grouped-term data are provided for fatigue/asthenia and the hematologic AEs (Supplementary Appendix). The time to onset, duration and management of the first episode of these AEs were analyzed, as well as the management and outcome of all episodes of these events.

### 2.4. Statistical analysis

As previously reported [6], SOLO1 was powered to detect differences in PFS.

Safety data were summarized in the safety analysis set (i.e. all randomized patients who received at least one dose of study treatment) and were summarized descriptively with no formal statistical analyses performed.

## 3. Results

Between September 3, 2013 and March 6, 2015, 391 patients were randomized, with 260 assigned to olaparib and 131 to placebo (Fig. S1). The safety analysis set comprised 260 olaparib and 130 placebo patients (one patient randomized to placebo withdrew before receiving study treatment). The date of data cut-off (DCO) for the primary analysis was May 17, 2018.

As previously reported, baseline characteristics were well balanced between treatment groups (Table 1) [6]. Nausea, asthenia, neutropenia and thrombocytopenia (all grades) were reported in few patients at baseline (Table 1). At baseline, fatigue was reported in 16.5% of olaparib patients and 19.8% of placebo patients, and anemia was reported in 19.2% and 10.7%, respectively.

The median (interquartile range [IQR]) duration of follow-up for the primary efficacy analysis was 40.7 months (34.9–42.9) for olaparib and 41.2 months (32.2–41.6) for placebo and the median (IQR) total duration of treatment was 24.6 months (11.2–24.9) for olaparib (consistent with the 2-year treatment cap) and 13.9 months (8.0–24.8) for placebo (consistent with the median PFS of 13.8 months in the placebo group). Treatment continued for at least 2 years in 57.3% of olaparib patients (47.3% completed 2 years' treatment and 10.0% continued treatment beyond 2 years) and 29.2% of placebo patients (26.9% completed 2 years' treatment and 2.3% continued treatment beyond 2 years).

At the primary DCO, 47.3% of patients in the olaparib group and 26.9% of patients in the placebo group had completed 2 years of maintenance therapy per protocol, 47.7% and 72.3%, respectively, had discontinued maintenance therapy for a reason other than the protocol-defined 2-year stopping rule and 5.0% and 0.8%, respectively, were still receiving maintenance therapy (Fig. S1). Reasons for discontinuation other than the 2-year stopping rule included disease progression (19.6% of olaparib patients vs 60.0% of placebo patients), adverse events (11.5% vs 2.3%), patient decision (8.5% vs 1.5%) and other reasons (8.1% vs 8.5%).

The most common AEs (all grades) were nausea, fatigue/asthenia, vomiting, anemia and diarrhea (Supplementary Table S1). AEs were predominantly grade 1–2, apart from anemia, which was the most common grade ≥3 AE (Supplementary Table S1).

Serious AEs occurred in 20.8% of olaparib patients and 12.3% of placebo patients; anemia was the most common serious AE (6.9% vs 0%) (Supplementary Table S2).

No AEs that occurred during administration of olaparib or placebo or up to 30 days after discontinuation of olaparib or placebo resulted in death.

**Fig. 1.**
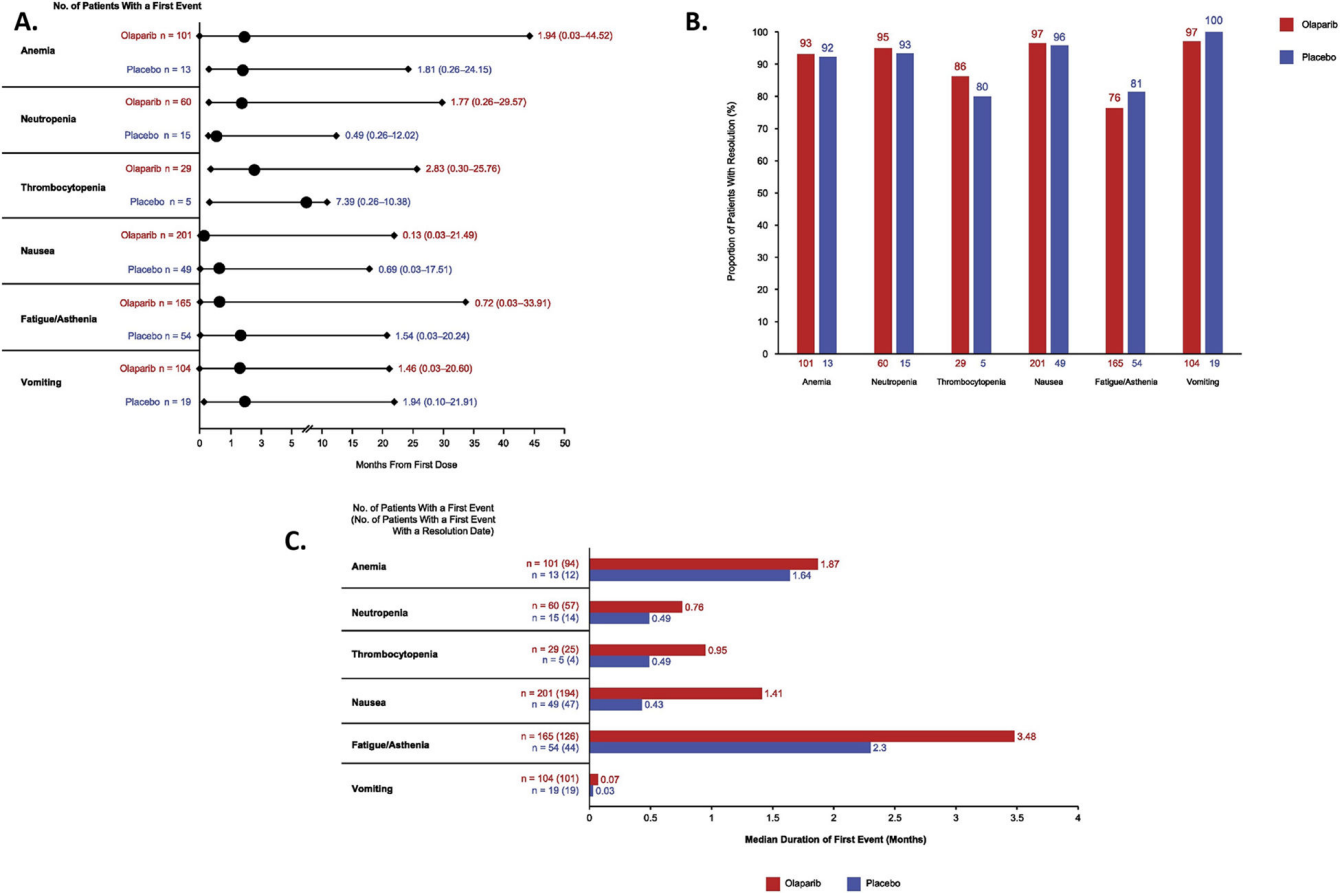
First occurrence of the most commonly reported hematologic and non-hematologic adverse events. Panel A shows the median time to first event. Circles represent medians, bars represent ranges. Panel B shows the proportion of patients with a first event with a resolution date; resolution was determined by the investigator. Percentages were calculated from the number of patients with a first event (shown below the bars) and take into account the date of data cut-off and the events that had a resolution date. Panel C shows the median duration of the first event. Adverse events with no end date were censored at the end of the safety follow-up or at data cut-off, as applicable.

The most common hematologic AEs were anemia, neutropenia and thrombocytopenia (Supplementary Table S1), with a median time to first onset (any grade) of 1.94, 1.77 and 2.83 months, respectively, for olaparib (Fig. 1A). For olaparib, resolution of the first event of anemia, neutropenia and thrombocytopenia occurred in the vast majority of patients experiencing these AEs (Fig. 1B), with the first event lasting a median of 1.87, 0.76 and 0.95 months, respectively (Fig. 1C). Olaparib dose reduction occurred in 43.6%, 15.8% and 16.0% of patients with resolution of anemia, neutropenia and thrombocytopenia, respectively (Supplementary Table S3).

**Fig. 2.**
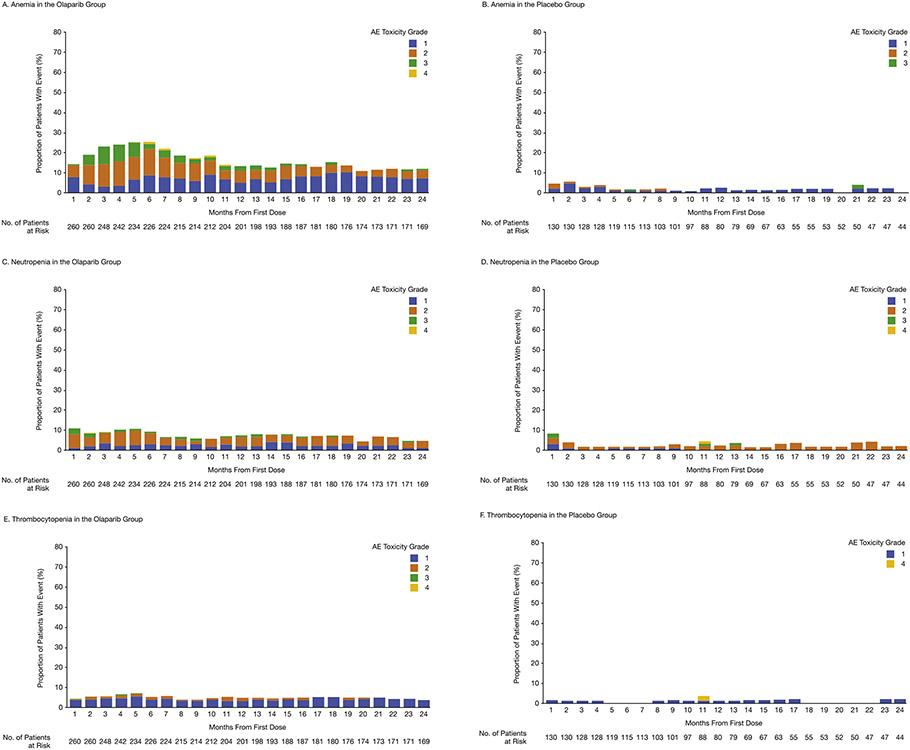

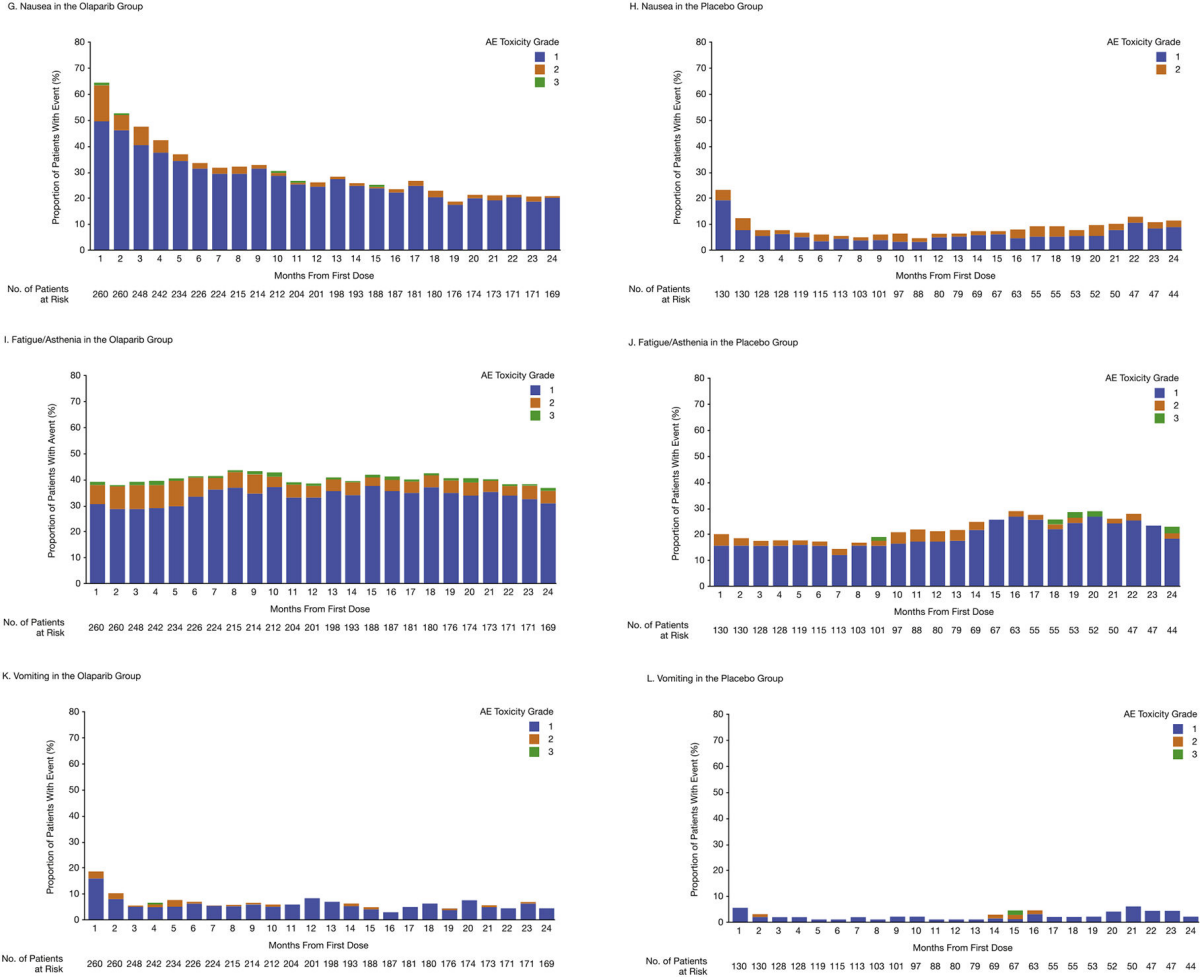
Prevalence by month and grade for the most common adverse events. Adverse events were graded according to the National Cancer Institute Common Terminology Criteria for Adverse Events, version 4.0. The number of patients at risk is the number of patients at each time point who were receiving olaparib or placebo or who were in safety follow-up to 30 days after the end of treatment. AE, adverse event.

For olaparib, the prevalence of anemia peaked at 6 months, with a reduction in the prevalence of grade 2 or worse anemia over time (Fig. 2A), and the prevalence of neutropenia (Fig. 2C) and thrombocytopenia (Fig. 2E) remained low; thrombocytopenia was predominantly grade 1 and neutropenia was predominantly grade 2 or higher. In the placebo group, the prevalence of hematologic AEs over time was low (Fig. 2B, D and F).

Overall, the median number of events per patient receiving olaparib was 1.0 for anemia and neutropenia and 2.0 for thrombocytopenia (Table 2). These AEs were usually managed with supportive treatment or dose modification, with few patients discontinuing olaparib (Table 2). At least one blood transfusion was administered to 60.4% of patients with anemia in the olaparib group and 23.1% of patients with anemia in the placebo group (Supplementary Appendix).

Most of the patients in the olaparib group with anemia, neutropenia or thrombocytopenia experienced recovery or resolution of the AE (Table 2).

Nausea, fatigue/asthenia and vomiting, the most common non-hematologic AEs in SOLO1, were predominantly grade 1 or 2 (Supplementary Table S1). For olaparib, the median time to first onset of nausea, fatigue/asthenia and vomiting of any grade was 0.13, 0.72 and 1.46 months, respectively (Fig. 1A). Resolution of the first event of nausea or vomiting occurred in >90% of olaparib patients experiencing these AEs (Fig. 1B), with the first event lasting a median of 1.41 and 0.07 months, respectively (Fig. 1C). The first event of fatigue/asthenia resolved in 76.4% of olaparib patients (Fig. 1B), with the first occurrence lasting a median of 3.48 months (Fig. 1C). Few olaparib patients required dose reduction to manage the first event of nausea, fatigue/asthenia or vomiting (Supplementary Table S3).

Nausea was the most common AE in the first month of maintenance olaparib; however, its prevalence and severity decreased rapidly (Fig. 2G). Although the overall prevalence of fatigue/asthenia appeared stable over time in the olaparib group, grade 2 or worse fatigue/asthenia decreased over time (Fig. 2I). The prevalence of vomiting, which was predominantly grade 1, remained low over time (Fig. 2K). In the placebo group, the prevalence of nausea (Fig. 2H) and vomiting (Fig. 2J) over time was low, with an apparent increase in the prevalence of fatigue/asthenia at 14 months (Fig. 2L).

The median number of events per patient in the olaparib group was 1.0 for nausea, fatigue/asthenia and vomiting (Table 2). These AEs were usually managed with supportive treatment or dose modification, with few patients discontinuing olaparib (Table 2). Propulsives (most commonly metoclopramide) were administered to 32.7% of olaparib patients versus 13.7% of placebo patients and serotonin 5-HT_3_ receptor antagonists were administered to 23.8% versus 16.0%, respectively (as reported on electronic case report forms) (Supplementary Appendix).

Most of the patients in the olaparib group with nausea, fatigue/asthenia or vomiting experienced recovery or resolution of the AE (Table 2).

Clinical chemistry results did not identify any new safety concerns. No clinically significant changes from baseline in clinical chemistry parameters (including albumin, alanine aminotransferase, aspartate aminotransferase, alkaline phosphatase, gamma glutamyltransferase and bilirubin) occurred in the olaparib or placebo groups. An increased blood creatinine level was reported as an AE in 8.1% of patients in the olaparib group and in 1.5% of patients in the placebo group; all increases in blood creatinine were grade 1 and none resulted in study drug discontinuation.

**Fig. 3.**
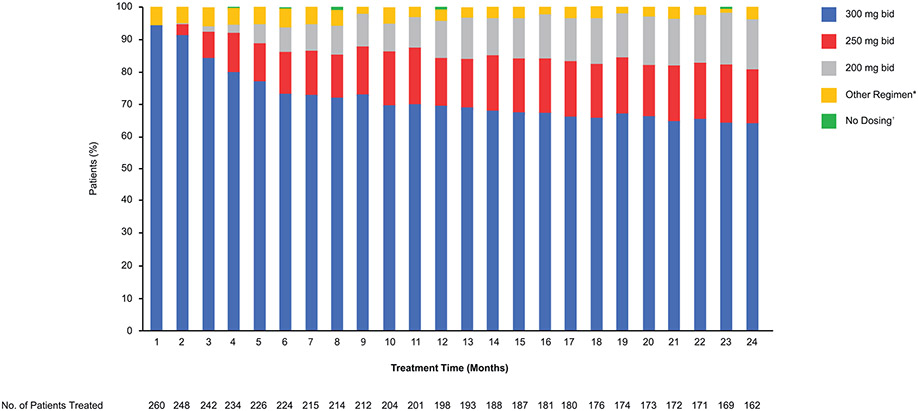
Olaparib dose reductions in SOLO1 over time. Number of patients treated at the start of each month. *’Other Regimen’ includes 150 mg qd, 150 mg bid, 200 mg qd, 250 mg qd, 300 mg qd and 450 mg bid. ^†^The category of ‘no dosing’ was assigned if the patient had dosing interrupted for the entire month window. bid, twice daily; qd, once daily.

Overall, AEs led to dose interruption in 51.9% of olaparib patients versus 16.9% of placebo patients, dose reduction in 28.5% versus 3.1%, respectively, and study drug discontinuation in 11.5% versus 2.3%, respectively. The median (IQR) duration of dose interruption because of AEs was 15.5 days (7–36) in the olaparib group and 13 days (7–17) in the placebo group. Of the 162 patients still receiving olaparib at month 24, 104 (64.2%) were receiving the recommended starting dose of olaparib 300 mg twice daily (Fig. 3). For olaparib, the most common AEs leading to dose reduction were anemia, fatigue, nausea and neutropenia (Supplementary Table S5). The most common AEs leading to study drug discontinuation were nausea (2.3% of olaparib patients vs 0.8% of placebo patients), anemia (2.3% vs 0%) and fatigue/asthenia (2.3% vs 0.8%).

MDS/AML, new primary malignancies and pneumonitis/interstitial lung disease (ILD) are AEs of interest for olaparib. AML was reported in three (1.2%) olaparib patients (Table 3), with all three cases resulting in death; no cases of MDS/AML were reported for placebo. Because death occurred >30 days after discontinuation of olaparib, these AML cases were not classified as AEs resulting in death. Following the primary analysis DCO, no new cases of MDS/AML were reported in either treatment group during longer-term follow-up (total median [IQR] duration of follow-up of 58.1 months [33.8–64.1] for olaparib and 59.6 months [30.8–63.5] for placebo) (DCO March 5, 2020).

New primary malignancies (excluding MDS/AML) had been reported in a total of seven (2.7%) olaparib patients and five (3.8%) placebo patients at the March 5, 2020 DCO (Supplementary Appendix).

Pneumonitis/ILD occurred in five (1.9%) of 260 patients in the olaparib group and no patients in the placebo group (Supplementary Appendix).

## 4. Discussion

In SOLO1, maintenance olaparib was associated with an unprecedented PFS benefit in patients with newly diagnosed advanced ovarian cancer and a BRCAm [6], and represents a new standard of care in this population [10]. Maintenance therapy with olaparib was capped at 2 years, meaning some patients were able to live progression-free for several years without treatment and its associated AEs [6,7]. To our knowledge, we report here the first detailed safety data for PARP inhibitor maintenance therapy in the newly diagnosed setting.

No new safety signals were identified and AEs were mostly mild to moderate, with anemia being the most common grade ≥3 AE. Anemia, neutropenia, thrombocytopenia, nausea, fatigue/asthenia and vomiting usually occurred early, although the peak in anemia prevalence at 6 months for olaparib is slightly later than previously reported in the relapsed disease setting [11]. The prevalence of fatigue/asthenia remained relatively constant throughout the olaparib treatment period; 29% of patients with fatigue/asthenia did not recover and other patients may have experienced recurrent episodes. The apparent increase in fatigue/asthenia with placebo at 14 months may reflect the impact of disease relapse (median PFS of 13.8 months in the placebo group versus 56.0 months in the olaparib group) [7]. Anemia, neutropenia, thrombocytopenia, nausea and vomiting were usually manageable with supportive therapy and/or dose modification.

Strict monitoring for anemia is suggested at the beginning of olaparib maintenance therapy. Complete blood counts should be performed monthly for the first 12 months, with periodic monitoring recommended thereafter [12]. Hematologic AEs should be managed with olaparib dose modification and, where appropriate, blood transfusion [12,13]. It may also be prudent to check folate levels in patients with anemia, as severe folate deficiency contributing to anemia was observed in a small number of patients receiving olaparib in the relapsed disease setting; administering folate supplements ameliorated the requirement for transfusion and olaparib dose modification in one patient [14].

Interruption of maintenance olaparib is recommended for severe hematologic toxicity or blood transfusion dependence [12]; blood counts should be monitored weekly until recovery. Bone marrow and/or blood cytogenetic analyses are recommended in patients with persistently abnormal blood parameters 4 weeks after interruption of olaparib [12].

Nausea and vomiting are usually manageable with antinausea/antiemetic therapy and/or olaparib dose modification [12,15]. Although antinausea prophylaxis is not recommended when maintenance olaparib is first started, it should be used in patients who subsequently experience nausea. In most cases, antinausea prophylaxis can be stopped after the first 2–3 months of therapy.

Supportive care (e.g. strategies to conserve energy) and dose modification can be used to manage fatigue/asthenia [12,15]. Although the prevalence of fatigue/asthenia appeared stable over time with olaparib in SOLO1, it was of predominantly grade 1 severity and few patients required dose reduction or discontinuation. Other possible causes of fatigue (e.g. anemia or depression) should be excluded in patients with ongoing fatigue [12,15].

Few SOLO1 patients required discontinuation of maintenance olaparib because of anemia, neutropenia, thrombocytopenia, nausea, fatigue/asthenia or vomiting.

The increase in blood creatinine level seen in some patients receiving maintenance olaparib might be explained by inhibition of renal transporters such as OCT2, MATE1 and MATE2K by olaparib leading to inhibition of tubular secretion of creatinine, as increases in blood creatinine levels were found to be reversible after discontinuation of olaparib [16].

During the 24-month treatment period in SOLO1, the majority of patients (64%) still receiving treatment remained on the olaparib starting dose without requiring dose reduction, with 17% receiving a reduced olaparib dose of 250 mg twice daily.

In terms of AEs of special interest, it is reassuring that no new cases of MDS/AML were reported between the primary analysis DCO and the DCO at March 5, 2020, and the incidence of new primary malignancies remained balanced between the treatment groups after approximately 5 years of follow-up. MDS/AML also occurs in patients with ovarian cancer who have not been exposed to PARP inhibitors [17], with a background risk of MDS/AML associated with use of select DNA-damaging therapies (including platinum-based agents) in earlier lines of chemotherapy [17].

Limited data are available concerning the risk of pneumonitis/ILD in patients receiving PARP inhibitor maintenance therapy. Five cases of pneumonitis/ILD were reported in SOLO1. The clinical presentation of pneumonitis/ILD is variable; interruption of maintenance olaparib is recommended in patients with new or worsening respiratory symptoms or abnormal chest radiologic findings and prompt investigation is warranted [12]. Olaparib should be discontinued if drug-induced pneumonitis/ILD is confirmed; treatment with corticosteroids may be indicated if pneumonitis/ILD is severe or progresses despite treatment interruption [12,18].

Although similarities are evident in the tolerability profiles of the different PARP inhibitors, with olaparib, niraparib, rucaparib and veliparib all associated with nausea, vomiting, fatigue/asthenia and anemia [6,9,19-22], distinct differences are also observed. For example, the frequency and severity of hematologic AEs differs between PARP inhibitors. In SOLO1, grade ≥3 thrombocytopenia and neutropenia were reported in 0.8% and 8.5% of olaparib patients, respectively. In a recent phase III trial, grade ≥3 thrombocytopenia, decreased platelet count, neutropenia and decreased neutrophil count were reported in 28.7%, 13.0%, 12.8% and 7.6% of patients, respectively, with newly diagnosed, advanced ovarian cancer who received maintenance niraparib [21]. The increased risk of thrombocytopenia, particularly grade ≥3 thrombocytopenia, necessitates weekly monitoring of blood counts for the first month of maintenance niraparib [23,24], whereas only monthly monitoring is needed with olaparib [12].

In terms of non-hematologic AEs, the risk of hypertension, insomnia or anxiety was not increased with olaparib versus placebo in SOLO1 [6]. However, these AEs have been reported with the PARP inhibitor niraparib [19,21,23,24], with hypertension thought to be related to off-target inhibition of dopamine, serotonin and norepinephrine transporters [13,25]. There was also no increased risk of liver function test abnormalities with olaparib versus placebo in SOLO1 [6], whereas increased levels of alanine and aspartate aminotransferase, mostly transient and self-limiting, have been reported with rucaparib in the relapsed disease setting [20]. To date, pneumonitis/ILD has mainly been reported, albeit rarely, with olaparib [6].

Strategies to mitigate for AEs with niraparib include starting at a lower dosage of 200 mg once daily, rather than the recommended starting dosage of 300 mg once daily [23,24], in patients with a low baseline bodyweight or platelet count [26]. Grade ≥3 thrombocytopenia and decreased platelet count were reported in 14.8% and 7.1% of patients, respectively, who started maintenance niraparib at 200 mg once daily following a protocol amendment in a phase III trial [21]. SOLO1 demonstrates that maintenance olaparib can be dosed over the long term in the first-line setting, with the majority of patients remaining on the starting dose and schedule, which supports the recommendation to start patients on an olaparib dosage of 300 mg twice daily [1,2].

## 5. Conclusions

Maintenance olaparib provided a substantial PFS benefit in patients with newly diagnosed advanced ovarian cancer and a BRCAm in SOLO1. Maintenance olaparib had manageable toxicity, with no new safety signals identified. The most commonly reported non-hematologic and hematologic AEs usually occurred early. Of 162 patients still receiving olaparib at month 24, 64.2% were receiving the recommended starting dose of olaparib 300 mg twice daily without requiring a dose reduction, with 17% receiving a reduced olaparib dose of 250 mg twice daily.

## Supplementary Material

1

## Figures and Tables

**Table 1:** Patient baseline characteristics.

Characteristic	Olaparib (N = 260)	Placebo (N = 131)
Response after platinum-based chemotherapy, n (%)		
Clinical complete response^a^	213 (81.9)	107 (81.7)
Clinical partial response^b^	47 (18.1)	24 (18.3)
ECOG performance status, n (%)		
0	200 (76.9)	105 (80.2)
1	60 (23.1)	25 (19.1)
Missing	0	1 (0.8)
Primary tumor location, n (%)		
Ovary	220 (84.6)	113 (86.3)
Fallopian tubes	22 (8.5)	11 (8.4)
Primary peritoneal	15 (5.8)	7 (5.3)
Other^c^	3 (1.2)	0
FIGO stage, n (%)		
III	220 (84.6)	105 (80.2)
IV	40 (15.4)	26 (19.8)
Histology, n (%)		
Serous	246 (94.6)	130 (99.2)
Endometrioid	9 (3.5)	0
Mixed serous/endometrioid	5 (1.9)	1 (0.8)
BRCA mutation,^d^ n (%)		
*BRCA1*	191 (73.5)	91 (69.5)
*BRCA2*	66 (25.4)	40 (30.5)
Both *BRCA1* and *BRCA2*	3 (1.2)	0
Adverse events at baseline,^e^ n (%)		
Nausea	15 (5.8)	9 (6.9)
Fatigue	43 (16.5)	26 (19.8)
Asthenia	12 (4.6)	4 (3.1)
Vomiting	0	1 (0.8)
Anemia^f^	50 (19.2)	14 (10.7)
Neutropenia^f^	2 (0.8)	4 (3.1)
Thrombocytopenia^f^	1 (0.4)	0

ECOG, Eastern Cooperative Oncology Group; FIGO, International Federation of Gynecology and Obstetrics; MedDRA, Medical Dictionary for Regulatory Activities; RECIST, Response Evaluation Criteria in Solid Tumors.

a Clinical complete response was defined as no evidence of disease on the post-treatment scan (according to modified RECIST, version 1.1) after chemotherapy and a normal CA-125 level.

b Partial response was defined as a ≥30% reduction in tumor volume from the start to the end of chemotherapy or no evidence of disease on the post-treatment scan, but a CA-125 level above the upper limit of normal.

c Other tumor locations included a combination of the ovary, fallopian tube, peritoneum, and omentum (n = 1), a combination of the ovary and peritoneum (n = 1), and a combination of the ovary and fallopian tube (n = 1).

d BRCA mutation status was determined centrally or locally.

e Adverse events recorded by investigators on the electronic case report form at baseline (MedDRA preferred term).

f Grade was not recorded, although at study entry, patients were required to have hemoglobin of ≥10.0 g/dL with no blood transfusion in the past 28 days, an absolute neutrophil count of ≥1.5 × 10^9^/L, and a platelet count of ≥100 × 10^9^/L (Supplementary Appendix).

**Table 2:** Management and outcome of the most commonly reported hematologic and non-hematologic adverse events.

Hematologic adverse events	Anemia^a^		Neutropenia^a^		Thrombocytopenia^a^	
	Olaparib (N = 260)	Placebo (N = 130)	Olaparib (N = 260)	Placebo (N = 130)	Olaparib (N = 260)	Placebo (N = 130)
Patients with event (all grades), n (%)	101 (38.8)	13 (10.0)	60 (23.1)	15 (11.5)	29 (11.2)	5 (3.8)
Median (range) number of adverse events per patient	1.00 (1–9)	1.00 (1–5)	1.00 (1–7)	1.00 (1–8)	2.00 (1–9)	1.00 (1–3)
Management, n (%)						
Supportive treatment	72 (27.7)	4 (3.1)	11 (4.2)	2 (1.5)	2 (0.8)	1 (0.8)
Dose interruption	58 (22.3)	1 (0.8)	30 (11.5)	5 (3.8)	6 (2.3)	0
Dose reduction	44 (16.9)	1 (0.8)	10 (3.8)	1 (0.8)	4 (1.5)	0
Discontinuation	6 (2.3)	0	1 (0.4)	0	1 (0.4)	0
Outcome, n (%)^b^						
Recovered/resolved	84 (83.2)	11 (84.6)	53 (88.3)	14 (93.3)	21 (72.4)	4 (80.0)
Recovered/resolved with sequelae	2 (2.0)	0	0	0	2 (6.9)	0
Recovering/resolving	5 (5.0)	0	1 (1.7)	0	0	0
Not recovered/resolved	10 (9.9)	2 (15.4)	6 (10.0)	1 (6.7)	6 (20.7)	1 (20.0)
Patients with grade ≥3 events, n (%)	56 (21.5)	2 (1.5)	22 (8.5)	6 (4.6)	2 (0.8)	2 (1.5)
Patients with serious events, n (%)	18 (6.9)	0	4 (1.5)	0	1 (0.4)	1 (0.8)
Non-hematologic adverse events	Nausea		Fatigue/asthenia^a^		Vomiting	
	Olaparib (N = 260)	Placebo (N = 130)	Olaparib (N = 260)	Placebo (N = 130)	Olaparib (N = 260)	Placebo (N = 130)
Patients with event (all grades), n (%)	201 (77.3)	49 (37.7)	165 (63.5)	54 (41.5)	104 (40.0)	19 (14.6)
Median (range) number of adverse events per patient	1.00 (1–14)	1.00 (1–6)	1.00 (1–8)	1.00 (1–3)	1.00 (1–12)	1.00 (1–5)
Management, n (%)						
Supportive treatment	117 (45.0)	15 (11.5)	11 (4.2)	0	28 (10.8)	3 (2.3)
Dose interruption	35 (13.5)	0	20 (7.7)	1 (0.8)	25 (9.6)	3 (2.3)
Dose reduction	10 (3.8)	0	15 (5.8)	1 (0.8)	0	0
Discontinuation	6 (2.3)	1 (0.8)	6 (2.3)	1 (0.8)	2 (0.8)	0
Outcome, n (%)^b^						
Recovered/resolved	183 (91.0)	46 (93.9)	103 (62.4)	41 (75.9)	100 (96.2)	19 (100.0)
Recovered/resolved with sequelae	1 (0.5)	0	1 (0.6)	1 (1.9)	1 (1.0)	0
Recovering/resolving	2 (1.0)	1 (2.0)	13 (7.9)	3 (5.6)	1 (1.0)	0
Not recovered/resolved	15 (7.5)	2 (4.1)	48 (29.1)	9 (16.7)	2 (1.9)	0
Patients with grade ≥3 events, n (%)	2 (0.8)	0	10 (3.8)	2 (1.5)	1 (0.4)	1 (0.8)
Patients with serious events, n (%)	0	0	0	0	0	1 (0.8)

Adverse events were graded according to the National Cancer Institute Common Terminology Criteria for Adverse Events, version 4.0. Adverse events were monitored throughout study treatment and for 30 days after discontinuation of study treatment.

a Grouped-term events.

b Percentages were calculated from the number of patients with that event.

**Table 3:** Summary of AML cases.^a^

Patient	Event	Patient age, years	BRCA mutation status	Duration of olaparib therapy, days	Reason for stopping olaparib	Time to AML diagnosis after stopping olaparib, days	Outcome
1	AML	52	*BRCA1* mutation	436	Persistent neutropenia and anemia	173	Fatal^b^
2	AML	52	*BRCA1* mutation	758	Completed 2 years' treatment	49	Fatal^b^
3	AML	64	*BRCA2* mutation	519	Dyspnea, pyrexia, and URTI with subsequent disease progression^c^	52	Fatal^b^

AML, acute myeloid leukemia; URTI, upper respiratory tract infection.

a All three patients had previously received six cycles of carboplatin plus paclitaxel. Cytogenetic abnormalities detected in these patients were: deletion in chromosome 7 or monosomy 7 (patient 1); deletion of the long arm of chromosome 5, with additional unidentified material on the short arm of chromosome 15 and the long arm of chromosome 21 and loss of chromosome 19 (patient 2); and loss of chromosome 7 (patient 3).

b In line with reporting standards for treatment-emergent adverse events, these cases of AML were not classified as adverse events resulting in death as death occurred >30 days after discontinuation of olaparib.

c This patient discontinued olaparib on day 519 because of dyspnea, pyrexia and URTI. Radiologic disease progression was detected on day 570.

## Data Availability

Data underlying the findings described in this manuscript may be obtained in accordance with AstraZeneca's data sharing policy described at https://astrazenecagrouptrials.pharmacm.com/ST/Submission/Disclosure.

## HIGHLIGHTS

- Detailed safety data from the SOLO1 trial of maintenance olaparib in newly diagnosed, advanced BRCA-mutated ovarian cancer.
- Maintenance olaparib had a predictable tolerability profile with no new safety signals identified.
- Adverse events usually occurred early, were largely manageable and led to discontinuation in a minority of patients.
- Risk of MDS/AML with maintenance olaparib in the newly diagnosed setting remained <1.5% with long-term follow-up of 5 years.
- The majority of patients were able to remain on the recommended starting dose of olaparib 300 mg twice daily.
