# Supplementary material for: Tolerability of maintenance olaparib in newly diagnosed patients with advanced ovarian cancer and a BRCA mutation in the randomized phase III SOLO1 trial
Source: Gynecol Oncol. Author manuscript; Available in PMC 2022 Oct 12. (PMC9555119; doi:10.1016/j.ygyno.2021.07.016)
Supplement: 1 [file NIHMS1835730-supplement-1.pdf]

Tolerability of maintenance olaparib in newly diagnosed patients with advanced ovarian cancer and a BRCA mutation in the randomized phase III SOLO1 trial

This appendix has been provided by the authors to give readers additional information about their work.

Supplement to: N. Colombo, K. Moore, G. Scambia, et al., Tolerability of maintenance olaparib in newly diagnosed patients with advanced ovarian cancer and a BRCA mutation in the randomized phase III SOLO1 trial

Contents

Tolerability of maintenance olaparib in newly diagnosed patients with advanced ovarian cancer and a BRCA mutation in the randomized phase III SOLO1 trial 1

SOLO1 investigators 2

1. Methods 4

1.1 Full eligibility criteria 4

1.2 Grading of non-hematologic adverse events 7

1.3 Dose modification and discontinuation for adverse events 8

1.4 Definitions for grouped-term hematologic adverse events 9

2. Results 10

2.1 Management of anemia 10

2.2 Management of nausea/vomiting 10

2.3 New primary malignancies 10

2.4 Pneumonitis/interstitial lung disease 11

Figure S1. Trial profile 13

Figure S2. Proportion of patients in the safety analysis set receiving blood transfusions by month 14

Table S1. Summary of adverse events[a] 15

Table S2. Summary of serious adverse events 16

Table S3. Management of the first occurrence of the most commonly reported hematologic and non-hematologic adverse events 17

Table S4. Administration of serotonin 5-HT_3 receptor antagonists by country 18

Table S5. Summary of adverse events leading to dose reduction[a] 18

Reference 18

SOLO1 investigators

The table below lists the principal investigators for each site who participated in the study.

Country	Principal investigators
Australia	Michael Friedlander, Linda Mileshkin, Clare Scott
Brazil	Sergio Azevedo, Giuliano Borges, Daniela Freitas, Gustavo Girotto, Roberto Hegg, Rodrigo Pereira, Geraldo Queiroz, Cristiano Souza
Canada	Allan Covens, Lucy Gilbert, Hal Hirte, Amit Oza, Marie Plante, Diane Provencher, Stephen Welch
China	Rutie Yin, Qi Zhou, Jianqing Zhu
France	Anne Floquet,* Florence Joly,* Marie-Christine Kaminsky,* Alexandra Leary,* Catherine Lhomme,*† Alain Lortholary,* Jean-Pierre Lotz,* Isabelle Ray-Coquard,* Frédéric Selle,*† Béatrice Weber*†
Israel	Amnon Amit, Ilan Bruchim, Ram Eitan, Ami Fishman, Moshe Inbar, Yfat Kadan, Roni Shapira Frommer
Italy	Francesco Cognetti, Nicoletta Colombo, PierFranco Conte, Vito Lorusso, Sandro Pignata, Francesco Raspagliesi, Giovanni Scambia, Paolo Scollo
Japan	Takayuki Enomoto, Keiichi Fujiwara, Yasuyuki Hirashima, Koji Matsumoto, Toshiaki Saito, Kazuhiro Takehara, Munetaka Takekuma, Kenji Tamura, Hidemichi Watari, Mayu Yunokawa
Netherlands	Roy Lalisang, Gabe Sonke
Poland	Mariusz Bidzinski, Tomasz Byrski, Maria Górnaś, Wojciech Rogowski, Pawel Rózanowski, Magdalena Sikorska, Anna Słowińska, Beata Śpiewankiewicz
Russia	Sergey Emelyanov, Larisa Kolomietc Petr Krivorotko, Alla Lisyanskaya, Olga Mikheeva, Galina Statsenko, Sergey Tyulyandin
South Korea	Chel Hun Choi, Byoung-Gie Kim, Jae Hoon Kim, Jae Weon Kim, Joo-Hyun Nam, Sang-Yoon Park, Sang-Young Ryu
Spain	Andrés Cervantes Ruipérez, Antonio González-Martín, Raúl Márquez Vázquez, Ana Oaknin, Beatriz Pardo Búrdalo, Andrés Poveda Velasco, Andrés Redondo, Ignacio Romero Noguera, María Jesús Rubio Pérez

United Kingdom	Susana Banerjee, James Brenton, Charlie Gourley, Jonathan Ledermann, Christopher Poole, Sarah Williams
United States	Sarah Adams, Carol Aghajanian, Daniel Anderson, Jeanne Anderson, Deborah Armstrong, Jamie Bakkum-Gamez, Joyce Barlin, Lisa Barroilhet, Kian Behbakht, Maria Bell, Katherine Bell-Mcguinn, Emily Berry, Stephanie Blank, Matthew Boente, William Bradley, Christopher Bryant, Thomas Buekers, Robert Burger, Michael Callahan, Guilherme Cantuaria, Michael Carney, Paul Celano, John Dalrymple, Christopher Darus, Susan Davidson, Oliver Dorigo, Gordon Downey, Babak Edraki, David Engle, Robin Farias-Eisner, John Farley, Lou Fehrenbacher, Mary Gordinier, Andrew Green, Parviz Hanjani, Charles Harrison, Monica Hayes, Robert Higgins, Joanie Hope, James Kendrick, Daniel Kredentser, Angela Kueck, Joseph Leach, Shashikant Lele, Timothy Lestingi, Joyce Liu, Joseph Lucci, Cara Mathews, Nathalie McKenzie, Donna McNamara, Joseph Merchant, Michael Method, David Miller, Susan Modesitt, Kathleen Moore, Peter Morris, Thomas Morrissey, David Mutch, David O'Malley, Janet Osborne, Dhimant Patel, Heather Pulaski, Elena Ratner, William Richards, Bobbie Jo Rimel, Luis Rojas-Espaillat, Peter Rose, Thomas Rutherford, Jeanne Schilder, Veronica Schimp, Angeles Alvarez Secord, Shohreh Shahabi, Mark Shahin, Gamini Soori, Nicola Spirtos, Robert Squatrito, Michael Stany, Frederick Stehman, Gregory Sutton, Nicholas Taylor, Meaghan Tenney, James Thigpen, Linda Van Le, Timothy Vanderkwaak, Sunitha Vemulapalli, Steven Waggoner, David Warshal, Robert Wenham, Shannon Westin, James Williams, Diane Yamada

*Groupe d'Investigateurs Nationaux pour l'Étude des Cancers Ovariens (GINECO), France; †Former principal investigator.

1. Methods

1.1 Full eligibility criteria

1.1.1 Inclusion criteria

1. Patients must be aged ≥18 years
2. Female patients with newly diagnosed, histologically confirmed, advanced (International Federation of Gynecology and Obstetrics [FIGO] stage III–IV) BRCA-mutated high-grade serous or high-grade endometrioid (based on local histopathologic findings) ovarian cancer, primary peritoneal cancer and/or fallopian tube cancer who have completed first-line, platinum-based chemotherapy (intravenous or intraperitoneal)
3. Stage III patients must have had one attempt at optimal debulking surgery (upfront or interval debulking). Stage IV patients must have had either a biopsy and/or upfront or interval debulking surgery
4. Documented mutation in *BRCA1* or *BRCA2* that is predicted to be deleterious or suspected deleterious (known or predicted to be detrimental/lead to loss of function)
5. Patients who have completed first-line platinum- (e.g. carboplatin or cisplatin) containing therapy (intravenous or intraperitoneal) prior to randomization
   - Patients must have, in the opinion of the investigator, clinical complete response or partial response and have no clinical evidence of disease progression on the post-treatment scan or a rising CA-125 level, following completion of this chemotherapy course. Patients with stable disease on the post-treatment scan at completion of first-line, platinum-containing therapy are not eligible for the study
   - ‘Response’ refers to patients being, in the opinion of the investigator, in clinical complete response or partial response on the post-treatment scan. Clinical complete response is defined as no evidence of Response Evaluation Criteria in Solid Tumors (RECIST) measurable or non-measurable disease on the post-treatment scan and a normal CA-125 level. Partial response is defined as ≥30% reduction in tumor volume demonstrated from the start to finish of chemotherapy OR no evidence of RECIST measurable disease on the post-treatment scan with a CA-125 level that has not decreased to within the normal range
   - Platinum-based chemotherapy course must have consisted of a minimum of six treatment cycles and a maximum of nine; however, if platinum-based therapy must be discontinued early as a result of toxicities specifically related to the platinum regimen, patients must have received a minimum of four cycles of the platinum regimen
   - Patients must not have received bevacizumab during their first-line course of treatment, either in combination or as maintenance therapy following combination therapy
   - Patients must not have received an investigational agent during their first-line course of chemotherapy
   - Patients must be randomized within 8 weeks after their last dose of chemotherapy (last dose is the day of the last infusion)

6. Pre-treatment CA-125 measurements must meet a criterion specified below:
   - If the first value is less than or equal to the upper limit of normal (ULN), the patient is eligible to be randomized and a second sample is not required
   - If the first value is greater than ULN, a second assessment must be performed at least 7 days after the first. If the second assessment is ≥15% more than the first, the patient is not eligible
7. Patients must have normal organ and bone marrow function measured within 28 days prior to administration of study treatment as defined below:
   - Hemoglobin (Hb) ≥10.0 g/dL with no blood transfusion in the past 28 days
   - Absolute neutrophil count ≥1.5 × 10^9/L
   - Platelet count ≥100 × 10^9/L
   - Total bilirubin ≤1.5 × institutional ULN
   - Aspartate aminotransferase/alanine aminotransferase ≤2.5 × institutional ULN unless liver metastases are present, in which case they must be ≤5 × ULN
   - Serum creatinine ≤1.5 × institutional ULN
8. Eastern Cooperative Oncology Group performance status 0–1
9. Patients must have a life expectancy ≥16 weeks
10. Postmenopausal or evidence of non-childbearing status for women of childbearing potential: negative urine or serum pregnancy test prior to Myriad BRCA test during screening part 1, within 28 days of study treatment and confirmed prior to treatment on day 1
11. Patient is willing and able to comply with the protocol for the duration of the study, including undergoing treatment and scheduled visits and examinations
12. Formalin-fixed, paraffin-embedded tumor sample from the primary cancer must be available for central testing. If there is not written confirmation of the availability of an archived tumor sample prior to enrolment, the patient is not eligible for the study

1.1.2 Exclusion criteria

1. Involvement in the planning and/or conduct of the study (applies to both AstraZeneca staff and/or staff at the study site)
2. *BRCA1* and/or *BRCA2* mutations that are considered to be non-detrimental
3. Patients with early-stage disease (FIGO stage I, IIA, IIB or IIC)
4. Stable disease or progressive disease on the post-treatment scan, or clinical evidence of progression at the end of the patient’s first-line chemotherapy treatment
5. Patients where more than one debulking surgery has been performed before randomization to the study. Patients are eligible who, at the time of diagnosis, are deemed to be unresectable and undergo only a biopsy or oophorectomy but then go on to receive chemotherapy and interval debulking surgery
6. Patients who have previously been diagnosed and treated for earlier-stage ovarian, fallopian tube or primary peritoneal cancer
7. Patients who have previously received chemotherapy for any abdominal or pelvic tumor, including treatment for prior diagnosis at an earlier stage for their ovarian, fallopian tube or primary peritoneal cancer. Patients who have

received prior adjuvant chemotherapy for localized breast cancer may be eligible, provided that it was completed more than 3 years prior to registration, and that the patient remains free of recurrent or metastatic disease

8. Patients with synchronous primary endometrial cancer unless both of the following criteria are met:
   - Stage <2
   - Less than 60 years old at the time of diagnosis of endometrial cancer with stage IA or IB grade 1 or 2, or stage IA grade 3 endometrioid adenocarcinoma, or ≥60 years old at the time of diagnosis of endometrial cancer with stage IA grade 1 or 2 endometrioid adenocarcinoma. Patients with serous or clear cell adenocarcinoma or carcinosarcoma of the endometrium are not eligible
9. Patients who have had drainage of their ascites during the final two cycles of their last chemotherapy regimen prior to enrolment on the study
10. Previous randomization in the present study
11. Participation in another clinical study with an investigational product during their chemotherapy course immediately prior to randomization
12. Any previous treatment with poly(ADP-ribose) polymerase (PARP) inhibitor, including olaparib
13. Other malignancy within the last 5 years, except adequately treated non-melanoma skin cancer; curatively treated *in situ* cancer of the cervix; ductal carcinoma *in situ*; stage I, grade 1 endometrial carcinoma; or other solid tumors, including lymphomas (without bone marrow involvement) curatively treated with no evidence of disease for ≥5 years. Patients with a history of localized breast cancer may be eligible, provided they completed their adjuvant chemotherapy more than 3 years prior to registration, and that the patient remains free of recurrent or metastatic disease
14. Resting electrocardiogram with a corrected QT interval >470 msec on two or more time points within a 24-hour period or family history of long QT syndrome
15. Patients receiving any systemic chemotherapy or radiotherapy (except for palliative reasons) within 3 weeks prior to study treatment (or a longer period depending on the defined characteristics of the agents used)
16. Concomitant use of known potent cytochrome P450 3A4 inhibitors, such as ketoconazole, itraconazole, ritonavir, indinavir, saquinavir, telithromycin, clarithromycin and nelfinavir
17. Persistent toxicities (Common Terminology Criteria for Adverse Events [CTCAE] grade ≥2) caused by previous cancer therapy, excluding alopecia
18. Patients with myelodysplastic syndromes/acute myeloid leukemia
19. Patients with symptomatic uncontrolled brain metastases. A scan to confirm the absence of brain metastases is not required. The patient can receive a stable dose of corticosteroids before and during the study, as long as these were started at least 4 weeks prior to treatment. Patients with spinal cord compression unless considered to have received definitive treatment for this and evidence of clinically stable disease for 28 days
20. Major surgery within 2 weeks of starting study treatment, and patients must have recovered from any effects of any major surgery

21. Patients considered a poor medical risk due to a serious, uncontrolled medical disorder, non-malignant systemic disease, or active, uncontrolled infection. Examples include, but are not limited to, uncontrolled ventricular arrhythmia, recent (within 3 months) myocardial infarction, uncontrolled major seizure disorder, unstable spinal cord compression, superior vena cava syndrome, extensive interstitial bilateral lung disease on high-resolution computed tomography (CT) scan, or any psychiatric disorder that prohibits obtaining informed consent
22. Patients unable to swallow orally administered medication, and patients with gastrointestinal disorders likely to interfere with absorption of the study medication
23. Breastfeeding women
24. Immunocompromised patients (e.g. patients who are known to be serologically positive for HIV)
25. Patients with a known hypersensitivity to olaparib or any of the excipients of the product
26. Patients with known active hepatitis (i.e. hepatitis B or C) due to risk of transmitting the infection through blood or other body fluids
27. Previous allogeneic bone marrow transplant
28. Whole blood transfusions in the last 120 days prior to entry to the study

1.2 Grading of non-hematologic adverse events

Adverse events were graded using National Cancer Institute CTCAE version 4.0 [1].

<u>Nausea</u>

Grade 1: Loss of appetite without alteration in eating habits
Grade 2: Oral intake decreased without significant weight loss, dehydration or malnutrition
Grade 3: Inadequate caloric or fluid intake; tube feeding, total parenteral nutrition or hospitalization indicated

<u>Fatigue</u>

Grade 1: Fatigue relieved by rest
Grade 2: Fatigue relieved by rest; limiting instrumental activities of daily living
Grade 3: Fatigue relieved by rest; limiting self-care activities of daily living

<u>Vomiting</u>

Grade 1: 1–2 episodes (separated by 5 minutes) in 24 hours
Grade 2: 3–5 episodes (separated by 5 minutes) in 24 hours
Grade 3: ≥6 episodes (separated by 5 minutes) in 24 hours; tube feeding, total parenteral nutrition, or hospitalization indicated
Grade 4: Life-threatening consequences; urgent intervention indicated
Grade 5: Death

For those events without assigned CTCAE grades, such as asthenia, CTCAE criteria converting mild, moderate, and severe events into CTCAE grades were used.

1.3 Dose modification and discontinuation for adverse events

Any toxicity observed during study treatment could be managed by study treatment interruption if deemed appropriate by the investigator. Repeat dose interruptions were allowed as required for a maximum of 14 days on each occasion (or up to 28 days after discussion with the study physician). Study treatment was interrupted until the patient recovered completely or the toxicity reverted to CTCAE grade 1 or less. Where toxicity reoccurred following rechallenge with study treatment, and where further dose interruptions were considered inadequate for the management of toxicity, then the patient was considered for dose reduction or had to permanently discontinue study treatment. Once the study treatment dose was reduced it could not be re-escalated, even if the adverse event resolved. Treatment must be interrupted if any CTCAE grade 3 or 4 adverse event occurred that the investigator considered to be related to administration of study treatment.

1.3.1 Management of anemia

For patients who developed anemia of National Cancer Institute CTCAE grade 3 (Hb <8 g/dL) or worse, study treatment should be interrupted for up to a maximum of 4 weeks to allow for bone marrow recovery and the patient should be managed appropriately. Study treatment could be restarted at the same dose if Hb recovered to >9 g/dL. Any subsequent anemia-related dose interruptions that were considered likely to be dose related or were coexistent with newly developed neutropenia and/or thrombocytopenia required study treatment dose reductions to 250 mg twice daily as a first step and to 200 mg twice daily as a second step.

If a patient had been treated for anemia with multiple blood transfusions without study treatment interruptions and became blood transfusion dependent (as judged by investigator), study treatment was interrupted for up to a maximum of 4 weeks to allow for bone marrow recovery, after which study treatment was restarted at a reduced dose.

1.3.2 Management of neutropenia and leukopenia

Study treatment should be interrupted if CTCAE grade 3 or worse neutropenia occurred. Study treatment was restarted at the same dose if neutropenia or leukopenia recovered up to CTCAE grade 1 or less (absolute neutrophil count $>1.5 \times 10^9$/L). Any subsequent dose interruptions required study treatment dose reductions to 250 mg twice daily as a first step and to 200 mg twice daily as a second step.

Primary prophylaxis with granulocyte colony-stimulating factor (G-CSF) was not recommended. However, if a patient developed febrile neutropenia, study treatment was stopped and appropriate management including G-CSF was given according to local hospital guidelines. G-CSF was not be used within at least 24 hours of the last dose of study treatment, and growth factor support was

stopped at least 24 hours before restarting the study drug (or at least 7 days before restarting the study drug for pegylated G-CSF).

1.3.3 Management of thrombocytopenia

Study treatment should be interrupted for a maximum of 4 weeks if CTCAE grade 3 or worse thrombocytopenia occurred.

1.3.4 Management of new or worsening pulmonary symptoms

If new or worsening pulmonary symptoms or radiologic abnormality occurred, study treatment should be interrupted and a diagnostic workup (including a high-resolution CT scan) should be performed to exclude pneumonitis. Following investigation, if no evidence of abnormality was observed on CT imaging and symptoms resolved, study treatment could be restarted, if deemed appropriate by the investigator.

1.4 Definitions for grouped-term hematologic adverse events

1.4.1 Anemia

Data for grouped-term anemia included patients with anemia, a decreased Hb level, decreased hematocrit, a decreased red cell count, erythropenia, macrocytic anemia, normochromic anemia, normochromic normocytic anemia or normocytic anemia.

1.4.2 Neutropenia

Data for grouped-term neutropenia included patients with neutropenia, febrile neutropenia, neutropenic sepsis, neutropenic infection, a decreased neutrophil count, idiopathic neutropenia, granulocytopenia, a decreased granulocyte count or agranulocytosis.

1.4.3 Thrombocytopenia

Data for grouped-term thrombocytopenia included patients with thrombocytopenia, decreased platelet production, decreased platelet count or decreased plateletcrit.

2. Results

2.1 Management of anemia

Among patients with anemia, at least one blood transfusion was administered to 61 (60.4%) of 101 patients in the olaparib group and three (23.1%) of 13 patients in the placebo group, and more than one blood transfusion was administered to 29 (28.7%) of 101 patients in the olaparib group and one (7.7%) of 13 patients in the placebo group.

In the safety analysis set, at least one blood transfusion was administered to 61 (23.5%) of 260 patients in the olaparib group and three (2.3%) of 130 patients in the placebo group, and more than one blood transfusion was administered to 29 (11.2%) of 260 patients in the olaparib group and one (0.7%) of 130 patients in the placebo group. In the safety analysis set, the majority of blood transfusions occurred within the first 4 months of study treatment (Figure S1).

Other anti-anemic preparations (e.g. erythropoiesis-stimulating agents) were administered in ten (3.8%) of 260 patients in the olaparib group and one (0.8%) of 131 patients in the placebo group. Of these, six patients (2.3%) in the olaparib arm and one patient (0.8%) in the placebo arms received epoetin alfa, two patients (0.8%) in the olaparib arm received darbepoetin alfa, and a further two patients (0.8%) in the olaparib arm received epoetin beta.

2.2 Management of nausea/vomiting

As reported on electronic case report forms, serotonin 5-HT_3 receptor antagonists were administered to 62 (23.8%) of 260 patients in the olaparib group and 21 (16.0%) of 131 patients in the placebo group. Administration of serotonin 5-HT_3 receptor antagonists by country is shown in Table S4.

2.3 New primary malignancies

New primary malignancies were reported in a total of seven (2.7%) of 260 patients in the olaparib group and five (3.8%) of 130 patients in the placebo group after a median (interquartile range) duration of follow-up of 58.1 (33.8–64.1) and 59.6 (30.8–63.5) months, respectively.

At the primary analysis data cut-off in the olaparib group:

- A 50-year-old woman (*BRCA1* mutation) had a grade 3 serious adverse event of invasive ductal breast carcinoma reported on day 329 of olaparib maintenance therapy. This patient discontinued maintenance olaparib on day 337. She recovered 513 days after onset of this adverse event, following surgical treatment and adjuvant chemotherapy.
- A 56-year-old woman (*BRCA1* mutation) had a grade 3 serious adverse event of breast cancer reported on day 55 of olaparib maintenance therapy. This patient discontinued maintenance olaparib on day 81. This adverse event was ongoing at the time of reporting.

- A 47-year-old woman (*BRCA1* mutation) had a grade 3 serious adverse event of intraductal proliferative breast lesion reported on day 257 of olaparib maintenance therapy. Olaparib maintenance therapy was interrupted on day 257 and restarted on day 262. This patient recovered 2 days after onset of this adverse event.
- A 63-year-old woman (*BRCA1* mutation; history of breast cancer 24 years previously) had a serious adverse event of oral cancer (grade 1 at onset) reported on day 576 of olaparib maintenance therapy. This patient discontinued maintenance olaparib on day 736 and recovered 219 days after onset of this adverse event.
- A 58-year-old woman (*BRCA1* mutation) had a serious adverse event of thyroid cancer (grade 2 at onset) reported on day 506 of olaparib maintenance therapy. She continued maintenance olaparib until day 758 (2-year treatment cap). This patient recovered 275 days after onset of this adverse event, following surgical treatment.

Following the primary analysis data cut-off in the olaparib group:

- A 44-year-old woman (*BRCA1* mutation) had breast cancer reported.
- A 47-year-old woman (*BRCA1* mutation) had invasive ductal carcinoma reported.

At the primary analysis data cut-off in the placebo group:

- A 59-year-old woman (*BRCA2* mutation) had a grade 2 serious adverse event of breast cancer reported on day 94 of placebo treatment. She discontinued placebo treatment on day 176 to initiate treatment with anastrozole, and this adverse event was ongoing when the patient died on day 788; her death was due to ovarian cancer.
- A 56-year-old woman (*BRCA1* mutation) had a grade 2 serious adverse event of breast cancer reported on day 240 of placebo treatment. She continued placebo treatment until day 247 when she discontinued due to disease progression. This adverse event was ongoing at the time of reporting.
- A 46-year-old woman (*BRCA1* mutation) had a grade 3 serious adverse event of breast cancer reported on day 666 of placebo treatment. She discontinued placebo treatment on day 667 and recovered 73 days after onset of this adverse event.

Following the primary analysis data cut-off in the olaparib group:

- A 49-year-old woman (*BRCA1* mutation) had lung adenocarcinoma reported.
- A 68-year-old woman (*BRCA1* mutation) had squamous cell carcinoma of the tongue reported.

2.4 Pneumonitis/interstitial lung disease

Four cases of pneumonitis and one case of interstitial lung disease (ILD) were reported in the olaparib group:

- A 51-year-old woman (an ex-smoker with a 30-year smoking history) presented with cough, dizziness, dyspnea, muscle weakness, anemia, and a decreased platelet count. Ground-glass opacities were seen on high-resolution CT scan with a grade 3, serious adverse event of pneumonitis reported on day 80 of olaparib maintenance therapy. The woman was treated with prednisolone and maintenance olaparib was discontinued on day 84. She recovered from pneumonitis 253 days after onset.
- A 61-year-old woman presented with shortness of breath, feeling lightheaded, and anemia. Diffuse ground-glass opacities were seen on CT scan with a grade 2, serious adverse event of pneumonitis reported on day 82 of maintenance olaparib. Olaparib maintenance therapy was interrupted from day 82 to 120, after which treatment resumed at a reduced dose. This patient recovered from pneumonitis 39 days after onset and continued on olaparib maintenance therapy.
- A 53-year-old woman presented with pyrexia. Ground-glass opacities were seen on CT scan with a grade 2, non-serious adverse event of pneumonitis reported on day 104 of olaparib maintenance therapy. The woman was treated with methylprednisolone and olaparib maintenance therapy was interrupted from day 105 to 124, after which maintenance olaparib was resumed at the same dose. She recovered from pneumonitis 21 days after onset and continued on olaparib maintenance therapy.
- A 60-year-old woman had a grade 1, non-serious adverse event of pneumonitis reported on day 81 of olaparib maintenance therapy. The woman discontinued maintenance olaparib on day 83 and was treated with levofloxacin. Pneumonitis was ongoing at the time of reporting.
- A 50-year-old woman (non-smoker) had a grade 1, non-serious adverse event of ILD reported on day 74 of olaparib maintenance therapy. Maintenance olaparib was interrupted on day 83 and discontinued on day 98. The woman was treated with ciprofloxacin, cough and cold preparations, and paracetamol, and recovered from ILD 101 days after onset.

Figure S1. Trial profile

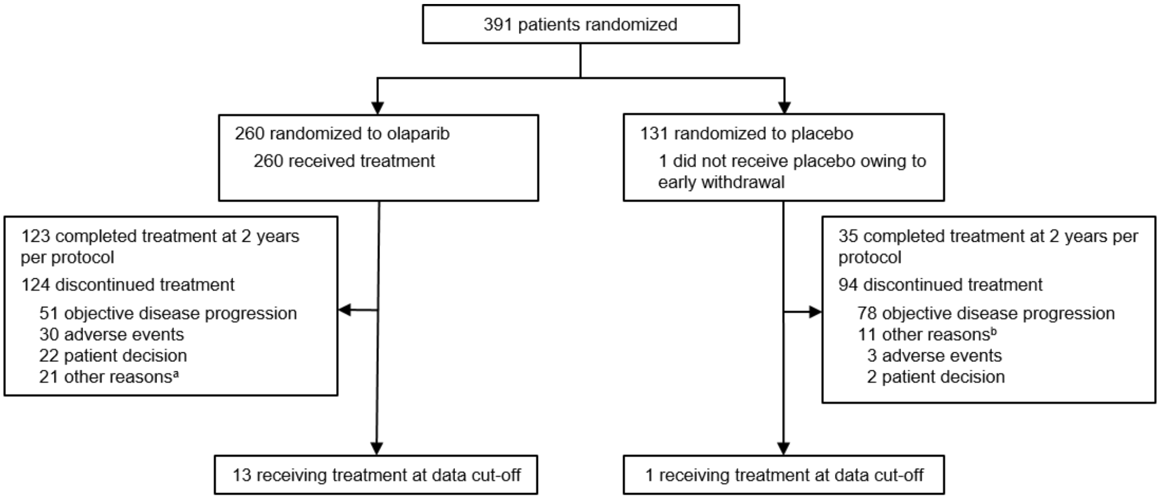

[a]Other reasons for discontinuation included study-specific discontinuation criteria (n = 6), severe protocol noncompliance (n = 3), unknown reason (n = 1) and other (n = 11)

[b]Other reasons for discontinuation included study-specific discontinuation criteria (n = 1), lost to follow-up (n = 1) and other (n = 9)

Figure S2. Proportion of patients in the safety analysis set receiving blood transfusions by month

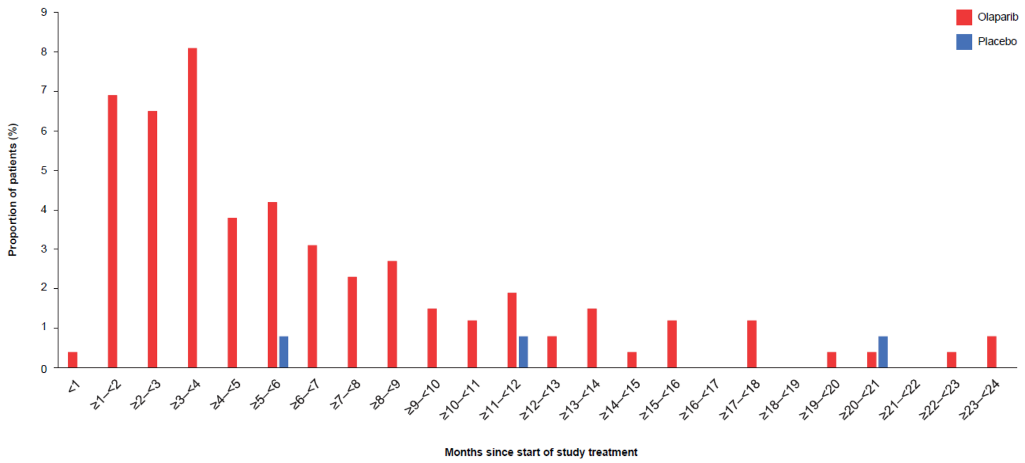

Safety analysis set included 260 patients in the olaparib arm and 130 patients in the placebo arm.

Table S1. Summary of adverse events[a]

Patients with adverse events, *n* (%)	Olaparib (*N* = 260)					Placebo (*N* = 130)				
	Total	G1	G2	G3	G4	Total	G1	G2	G3	G4
Any adverse event	256 (98.5)	34 (13.1)	120 (46.2)	93 (35.8)	9 (3.5)	120 (92.3)	35 (26.9)	61 (46.9)	21 (16.2)	3 (2.3)
Nausea	201 (77.3)	148 (56.9)	51 (19.6)	2 (0.8)	0	49 (37.7)	34 (26.2)	15 (11.5)	0	0
Fatigue/asthenia[b]	165 (63.5)	86 (33.1)	69 (26.5)	10 (3.8)	0	54 (41.5)	38 (29.2)	14 (10.8)	2 (1.5)	0
Vomiting	104 (40.0)	79 (30.4)	24 (9.2)	1 (0.4)	0	19 (14.6)	16 (12.3)	2 (1.5)	1 (0.8)	0
Anemia[c]	101 (38.8)	17 (6.5)	28 (10.8)	50 (19.2)	6 (2.3)	13 (10.0)	6 (4.6)	5 (3.8)	2 (1.5)	0
Diarrhea	89 (34.2)	61 (23.5)	20 (7.7)	8 (3.1)	0	32 (24.6)	26 (20.0)	6 (4.6)	0	0
Constipation	72 (27.7)	64 (24.6)	8 (3.1)	0	0	25 (19.2)	19 (14.6)	6 (4.6)	0	0
Dysgeusia	68 (26.2)	62 (23.8)	6 (2.3)	0	0	5 (3.8)	5 (3.8)	0	0	0
Arthralgia	66 (25.4)	55 (21.2)	10 (3.8)	0	0	35 (26.9)	28 (21.5)	7 (5.4)	0	0
Abdominal pain	64 (24.6)	50 (19.2)	10 (3.8)	4 (1.5)	0	25 (19.2)	16 (12.3)	8 (6.2)	1 (0.8)	0
Neutropenia[d]	60 (23.1)	9 (3.5)	29 (11.2)	21 (8.1)	1 (0.4)	15 (11.5)	4 (3.1)	5 (3.8)	5 (3.8)	1 (0.8)
Headache	59 (22.7)	47 (18.1)	11 (4.2)	1 (0.4)	0	31 (23.8)	25 (19.2)	3 (2.3)	3 (2.3)	0
Dizziness	51 (19.6)	48 (18.5)	3 (1.2)	0	0	20 (15.4)	18 (13.8)	1 (0.8)	1 (0.8)	0
Decreased appetite	51 (19.6)	45 (17.3)	6 (2.3)	0	0	13 (10.0)	11 (8.5)	2 (1.5)	0	0
Upper abdominal pain	46 (17.7)	41 (15.8)	5 (1.9)	0	0	17 (13.1)	13 (10.0)	4 (3.1)	0	0
Dyspepsia	43 (16.5)	31 (11.9)	12 (4.6)	0	0	16 (12.3)	12 (9.2)	4 (3.1)	0	0
Cough	42 (16.2)	33 (12.7)	9 (3.5)	0	0	28 (21.5)	24 (18.5)	4 (3.1)	0	0
Back pain	40 (15.4)	34 (13.1)	6 (2.3)	0	0	16 (12.3)	14 (10.8)	2 (1.5)	0	0
Dyspnea	39 (15.0)	27 (10.4)	12 (4.6)	0	0	7 (5.4)	5 (3.8)	2 (1.5)	0	0
Pyrexia	31 (11.9)	25 (9.6)	6 (2.3)	0	0	12 (9.2)	10 (7.7)	2 (1.5)	0	0
UTI	31 (11.9)	8 (3.1)	21 (8.1)	2 (0.8)	0	8 (6.2)	5 (3.8)	3 (2.3)	0	0
Thrombocytopenia[e]	29 (11.2)	17 (6.5)	10 (3.8)	1 (0.4)	1 (0.4)	5 (3.8)	3 (2.3)	0	0	2 (1.5)
Myalgia	28 (10.8)	23 (8.8)	5 (1.9)	0	0	13 (10.0)	11 (8.5)	2 (1.5)	0	0
URTI	28 (10.8)	16 (6.2)	12 (4.6)	0	0	12 (9.2)	8 (6.2)	4 (3.1)	0	0
Pain in extremity	28 (10.8)	20 (7.7)	8 (3.1)	0	0	11 (8.5)	8 (6.2)	3 (2.3)	0	0
Nasopharyngitis	27 (10.4)	24 (9.2)	3 (1.2)	0	0	17 (13.1)	16 (12.3)	1 (0.8)	0	0
Insomnia	27 (10.4)	20 (7.7)	7 (2.7)	0	0	16 (12.3)	13 (10.0)	3 (2.3)	0	0
Depression	13 (5.0)	7 (2.7)	5 (1.9)	1 (0.4)	0	13 (10.0)	9 (6.9)	3 (2.3)	1 (0.8)	0

[a]All grade and grade 1, 2, 3 and 4 adverse events are shown (there were no grade 5 events) throughout the study drug treatment period and up to 30 days after the end of treatment (National Cancer Institute Common Terminology Criteria for Adverse Events, version 4.0). Only adverse events occurring in at least 10.0% of patients (all grades) in either treatment group are shown.

[b]Grouped term. All grade and grade 1, grade 2 and grade 3 fatigue occurred in 106 (40.8%), 65 (25.0%), 36 (13.8%) and 5 (1.9%) olaparib patients, respectively, and in 39 (30.0%), 28 (21.5%), 9 (6.9%) and 2 (1.5%) placebo patients, respectively. All grade and grade 1, grade 2 and grade 3 asthenia occurred in 63 (24.2%), 24 (9.2%), 34 (13.1%) and 5 (1.9%) olaparib patients, respectively, and in 16 (12.3%), 10 (7.7%), 6 (4.6%) and 0 placebo patients, respectively.

[c]Anemia includes anemia, decreased hemoglobin level, decreased hematocrit, decreased red blood cell count, erythropenia, macrocytic anemia, normochromic anemia, normochromic normocytic anemia and normocytic anemia.

[d]Neutropenia includes neutropenia, febrile neutropenia, neutropenic sepsis, neutropenic infection, decreased neutrophil count, idiopathic neutropenia, granulocytopenia, decreased granulocyte count and agranulocytosis.

[e]Thrombocytopenia includes thrombocytopenia, decreased platelet production, decreased platelet count or decreased plateletcrit.

G, grade; URTI, upper respiratory tract infection; UTI, urinary tract infection.

Table S2. Summary of serious adverse events

Patients with serious adverse events, *n* (%)	Olaparib (*N* = 260)					Placebo (*N* = 130)				
	**Total**	**G1**	**G2**	**G3**	**G4**	**Total**	**G1**	**G2**	**G3**	**G4**
Anemia[a]	18 (6.9)	0	1 (0.4)	12 (4.6)	5 (1.9)	0	0	0	0	0
Neutropenia[a]	4 (1.5)	0	1 (0.4)	3 (1.2)	0	0	0	0	0	0
UTI	3 (1.6)	0	1 (0.4)	2 (0.8)	0	0	0	0	0	0
Viral infection	2 (0.8)	0	2 (0.8)	0	0	0	0	0	0	0
Syncope	2 (0.8)	1 (0.4)	0	1 (0.4)	0	0	0	0	0	0
Transient ischemic attack	2 (0.8)	1 (0.4)	1 (0.4)	0	0	0	0	0	0	0
Pneumonitis	2 (0.8)	0	1 (0.4)	1 (0.4)	0	0	0	0	0	0
Pulmonary embolism	2 (0.8)	0	0	2 (0.8)	0	0	0	0	0	0
Abdominal pain	2 (0.8)	0	0	2 (0.8)	0	1 (0.8)	0	0	1 (0.8)	0
Small intestinal obstruction	2 (0.8)	0	0	2 (0.8)	0	1 (0.8)	0	0	0	1 (0.8)
Rotator cuff syndrome	2 (0.8)	0	0	2 (0.8)	0	0	0	0	0	0
Cellulitis	1 (0.4)	0	0	1 (0.4)	0	1 (0.8)	0	0	1 (0.8)	0
Infected lymphocele	1 (0.4)	0	0	1 (0.4)	0	0	0	0	0	0
Medical device site cellulitis	1 (0.4)	0	0	1 (0.4)	0	0	0	0	0	0
URTI	1 (0.4)	0	1 (0.4)	0	0	0	0	0	0	0
Urosepsis	1 (0.4)	0	0	1 (0.4)	0	0	0	0	0	0
Sepsis	1 (0.4)	0	0	0	1 (0.4)	0	0	0	0	0
Breast cancer	1 (0.4)	0	0	1 (0.4)	0	3 (2.3)	0	1 (0.8)	2 (1.5)	0
Intraductal proliferative breast lesion	1 (0.4)	0	0	1 (0.4)	0	0	0	0	0	0
Invasive ductal breast carcinoma	1 (0.4)	0	0	1 (0.4)	0	0	0	0	0	0
Lip and/or oral cavity cancer	1 (0.4)	0	0	1 (0.4)	0	0	0	0	0	0
Thyroid cancer	1 (0.4)	0	0	1 (0.4)	0	0	0	0	0	0
Leukopenia[a]	1 (0.4)	0	0	1 (0.4)	0	0	0	0	0	0
Splenic cyst	1 (0.4)	0	0	1 (0.4)	0	0	0	0	0	0
Cell death	1 (0.4)	0	0	1 (0.4)	0	0	0	0	0	0
Depression	1 (0.4)	0	0	1 (0.4)	0	0	0	0	0	0
Ataxia	1 (0.4)	0	0	1 (0.4)	0	0	0	0	0	0
Headache	1 (0.4)	0	0	1 (0.4)	0	2 (1.5)	0	0	2 (1.5)	0
Peripheral neuropathy	1 (0.4)	0	0	1 (0.4)	0	0	0	0	0	0
Diarrhea	1 (0.4)	0	0	1 (0.4)	0	0	0	0	0	0
Ileus	1 (0.4)	0	0	1 (0.4)	0	0	0	0	0	0
Subileus	1 (0.4)	0	1 (0.4)	0	0	0	0	0	0	0
Intestinal obstruction	1 (0.4)	0	0	1 (0.4)	0	0	0	0	0	0
Acute cholecystitis	1 (0.4)	0	0	1 (0.4)	0	0	0	0	0	0
Chills	1 (0.4)	1 (0.4)	0	0	0	0	0	0	0	0
Incarcerated hernia	1 (0.4)	0	0	1 (0.4)	0	0	0	0	0	0
Pyrexia	1 (0.4)	1 (0.4)	0	0	0	0	0	0	0	0
Thrombocytopenia[a]	1 (0.4)	0	1 (0.4)	0	0	1 (0.8)	0	0	0	1 (0.8)
Stab wound	1 (0.4)	0	0	1 (0.4)	0	0	0	0	0	0
Wound complication	1 (0.4)	0	0	1 (0.4)	0	0	0	0	0	0
Appendicitis	0	0	0	0	0	1 (0.8)	0	0	1 (0.8)	0

Patients with serious adverse events, *n* (%)	Olaparib (*N* = 260)					Placebo (*N* = 130)				
	Total	G1	G2	G3	G4	Total	G1	G2	G3	G4
Cystitis	0	0	0	0	0	1 (0.8)	0	1 (0.8)	0	0
Pneumonia	0	0	0	0	0	1 (0.8)	0	0	1 (0.8)	0
Wound infection	0	0	0	0	0	1 (0.8)	0	0	1 (0.8)	0
Thyroid cancer recurrent	0	0	0	0	0	1 (0.8)	0	0	1 (0.8)	0
Lymphopenia[a]	0	0	0	0	0	1 (0.8)	0	0	1 (0.8)	0
Myocardial infarction	0	0	0	0	0	1 (0.8)	0	0	1 (0.8)	0
Incarcerated umbilical hernia	0	0	0	0	0	1 (0.8)	0	0	1 (0.8)	0
Umbilical hernia	0	0	0	0	0	1 (0.8)	0	1 (0.8)	0	0
Vomiting	0	0	0	0	0	1 (0.8)	0	0	1 (0.8)	0
Limb discomfort	0	0	0	0	0	1 (0.8)	0	1 (0.8)	0	0
ALT increased	0	0	0	0	0	1 (0.8)	0	0	1 (0.8)	0
Accidental exposure to product by child	0	0	0	0	0	1 (0.8)	1 (0.8)	0	0	0
Drug administration error	0	0	0	0	0	1 (0.8)	1 (0.8)	0	0	0
Thoracic vertebral fracture	0	0	0	0	0	1 (0.8)	0	1 (0.8)	0	0

[a]Grouped terms.
ALT, alanine aminotransferase; G, grade; URTI, upper respiratory tract infection; UTI, urinary tract infection.

Table S3. Management of the first occurrence of the most commonly reported hematologic and non-hematologic adverse events

**Hematologic adverse events, *n* (%)**	**Anemia[a]**		**Neutropenia[a]**		**Thrombocytopenia[a]**	
	**Olaparib (*N* = 260)**	**Placebo (*N* = 130)**	**Olaparib (*N* = 260)**	**Placebo (*N* = 130)**	**Olaparib (*N* = 260)**	**Placebo (*N* = 130)**
Patients with event (all grades)	101 (38.8)	13 (10.0)	60 (23.1)	15 (11.5)	29 (11.2)	5 (3.8)
Patients with a first event with a resolution date (all grades)[b]	94 (93.1)	12 (92.3)	57 (95.0)	14 (93.3)	25 (86.2)	4 (80.0)
Management of first event with a resolution date[c]						
Supportive treatment	68 (72.3)	4 (33.3)	11 (19.3)	2 (14.3)	2 (8.0)	1 (25.0)
Dose interruption	57 (60.6)	1 (8.3)	27 (47.4)	5 (35.7)	6 (24.0)	0
Dose reduction	41 (43.6)	1 (8.3)	9 (15.8)	1 (7.1)	4 (16.0)	0
Discontinuation	6 (6.4)	0	0	0	1 (4.0)	0
**Non-hematologic adverse events, *n* (%)**	**Nausea**		**Fatigue/asthenia[a]**		**Vomiting**	
	**Olaparib (*N* = 260)**	**Placebo (*N* = 130)**	**Olaparib (*N* = 260)**	**Placebo (*N* = 130)**	**Olaparib (*N* = 260)**	**Placebo (*N* = 130)**
Patients with event (all grades)	201 (77.3)	49 (37.7)	165 (63.5)	54 (41.5)	104 (40.0)	19 (14.6)
Patients with a first event with a resolution date (all grades)[b]	194 (96.5)	47 (95.9)	126 (76.4)	44 (81.5)	101 (97.1)	19 (100.0)
Management of first event with a resolution date[c]						
Supportive treatment	109 (56.2)	15 (31.9)	9 (7.1)	0	26 (25.7)	3 (15.8)
Dose interruption	32 (16.5)	0	14 (11.1)	1 (2.3)	23 (22.8)	3 (15.8)
Dose reduction	10 (5.2)	0	12 (9.5)	1 (2.3)	0	0
Discontinuation	3 (1.5)	1 (2.1)	4 (3.2)	1 (2.3)	1 (1.0)	0

[a]Grouped-term events.
[b]Number (%) of patients with a first event that has a resolution date. Percentages were calculated from the number of patients with an event.
[c]Percentages were calculated from the number of patients with a first event with a resolution date.

Table S4. Administration of serotonin 5-HT_3 receptor antagonists by country

Country	Olaparib (*N* = 260)	Placebo (*N* = 131)
All	62 (23.8)	21 (16.0)
USA	39 (15.0)	14 (10.7)
Canada	5 (1.9)	2 (1.5)
Italy	5 (1.9)	1 (0.8)
Korea	3 (1.2)	2 (1.5)
Australia	3 (1.2)	0
Spain	2 (0.8)	1 (0.8)
China	1 (0.4)	0
France	1 (0.4)	0
UK	1 (0.4)	0
Israel	1 (0.4)	0
Netherlands	1 (0.4)	0
Brazil	0	1 (0.8)

Table S5. Summary of adverse events leading to dose reduction[a]

Patients with adverse event leading to dose reduction, *n* (%)	Olaparib (*N* = 260)	Placebo (*N* = 130)
Any	74 (28.5)	4 (3.1)
Anemia	44 (16.9)	1 (0.8)
Fatigue	10 (3.8)	1 (0.8)
Nausea	10 (3.8)	0
Neutropenia	9 (3.5)	1 (0.8)
Asthenia	5 (1.9)	0
Leukopenia	4 (1.5)	0
Thrombocytopenia	3 (1.2)	0
Depression	2 (0.8)	0

[a]Adverse event leading to dose reduction in more than one patient in the olaparib group.

Reference

[1] US Department of Health and Human Services, Common Terminology Criteria for Adverse Events (CTCAE) version 4.0, https://evs.nci.nih.gov/ftp1/CTCAE/CTCAE_4.03/Archive/CTCAE_4.0_2009-05-29_QuickReference_8.5x11.pdf, 2009 (accessed 12 March 2020).
